# Decoding neuronal gene expression: integrative insights from omics and AI

**DOI:** 10.1186/s40708-026-00323-z

**Published:** 2026-07-21

**Authors:** Aranyak Goswami, Rushikesh R. Lagad, Shakil Rafi

**Affiliations:** Department of Animal Science, Dale Bumpers College of Agricultural, Food and Life Sciences, University of Arkansas, Fayetteville, AR 72701 USA

**Keywords:** Multi-omics integration, Brain informatics, Neuronal gene expression, Graph neural networks, Explainable AI, Spatial transcriptomics, Foundation models

## Abstract

Neuronal functional diversity and pathological vulnerability are governed by multi-layered regulatory programs. While high-throughput omics and neuroimaging provide high-resolution snapshots of these programs, bridging the gap between molecular dynamics and macro-scale brain architecture remains a significant informatics challenge. This review synthesizes the evolution of computational frameworks in neuro-omics—transitioning from descriptive co-expression modules to causal graph neural networks and cross-scale foundation models. We evaluate these methodologies within the context of Alzheimer’s disease, schizophrenia, and epilepsy, identifying critical bottlenecks in data harmonization, spatial alignment, and causal interpretability.

**Supplementary Information:**

The online version contains supplementary material available at 10.1186/s40708-026-00323-z.

## Introduction

The human brain’s operational complexity is predicated on a precisely orchestrated gene regulatory landscape across diverse cell types and developmental stages. The advent of single-cell multi-omics and spatial transcriptomics has generated datasets of unprecedented dimensionality, yet the “curse of dimensionality” and inherent biological noise necessitate robust analytical frameworks. Traditional statistical approaches often fail to capture the non-linear dependencies between epigenetic modifications, transcriptional output, and emergent neural phenotypes. Consequently, Artificial Intelligence (AI) has become indispensable for decoding these regulatory logics.

## A unifying framework for neuroinformatics

To navigate the expanding landscape of computational tools, it is necessary to view integrative pipelines as a series of complementary, functional layers rather than isolated techniques. This layered architecture reflects the field’s progression from classical linear statistics to multimodal AI systems.

Key datasets, computational techniques, and integration challenges are summarized in Supplementary Table S1 (Additional file 1), including major brain informatics resources and atlas-scale omics platforms [1–7]. This supplementary table is provided only as the external Additional file 1 and is not part of the main-text table numbering. The main manuscript table sequence is reserved for Table 1 (milestone neuro-omics AI methods) and Table 2 (comparative GRN inference routes).

### Technological evolution and functional architecture of the integrative pipeline

The field has progressed through three successive eras of increasing explanatory power. The statistical era established descriptive co-expression modules via WGCNA and regularized regression (LASSO, ElasticNet), providing a baseline for gene-module discovery. The deep learning era introduced non-linear representation learning through VAEs, scVI, and GNNs, enabling single-cell resolution and multimodal fusion. We now operate at the frontier of the causal and foundation era, characterized by causal GNNs, multimodal Transformers, and large-scale foundation models (scGPT, Geneformer) that support cross-scale prediction and mechanistic interpretation [8–14]. Table 1 summarizes milestone methods and technical breakthroughs across these eras.

**Table 1:** Milestone methods in neuro-omics AI

Era	Representative methods	Key technical breakthrough	Iterative logic
Statistical / co-expression	WGCNA, PCA, LASSO, ElasticNet	Descriptive gene modules; sparse biomarker selection	Identify co-regulated gene sets from bulk expression data
Probabilistic / graphical	Bayesian networks, HMMs, GENIE3	Directionality inference; latent state modeling	Add prior probability and hidden-state transitions
Deep learning	VAEs, scVI, GNNs, SCENIC, MOFA+	Non-linear representation; single-cell resolution	Learn compressed latent spaces capturing biological variation
Causal and foundation models	Causal GNNs, transformers, scGPT, geneformer	Cross-scale prediction; mechanistic hypotheses; zero-shot transfer	Unify modalities and enable in silico perturbation simulation

**Fig. 1:**
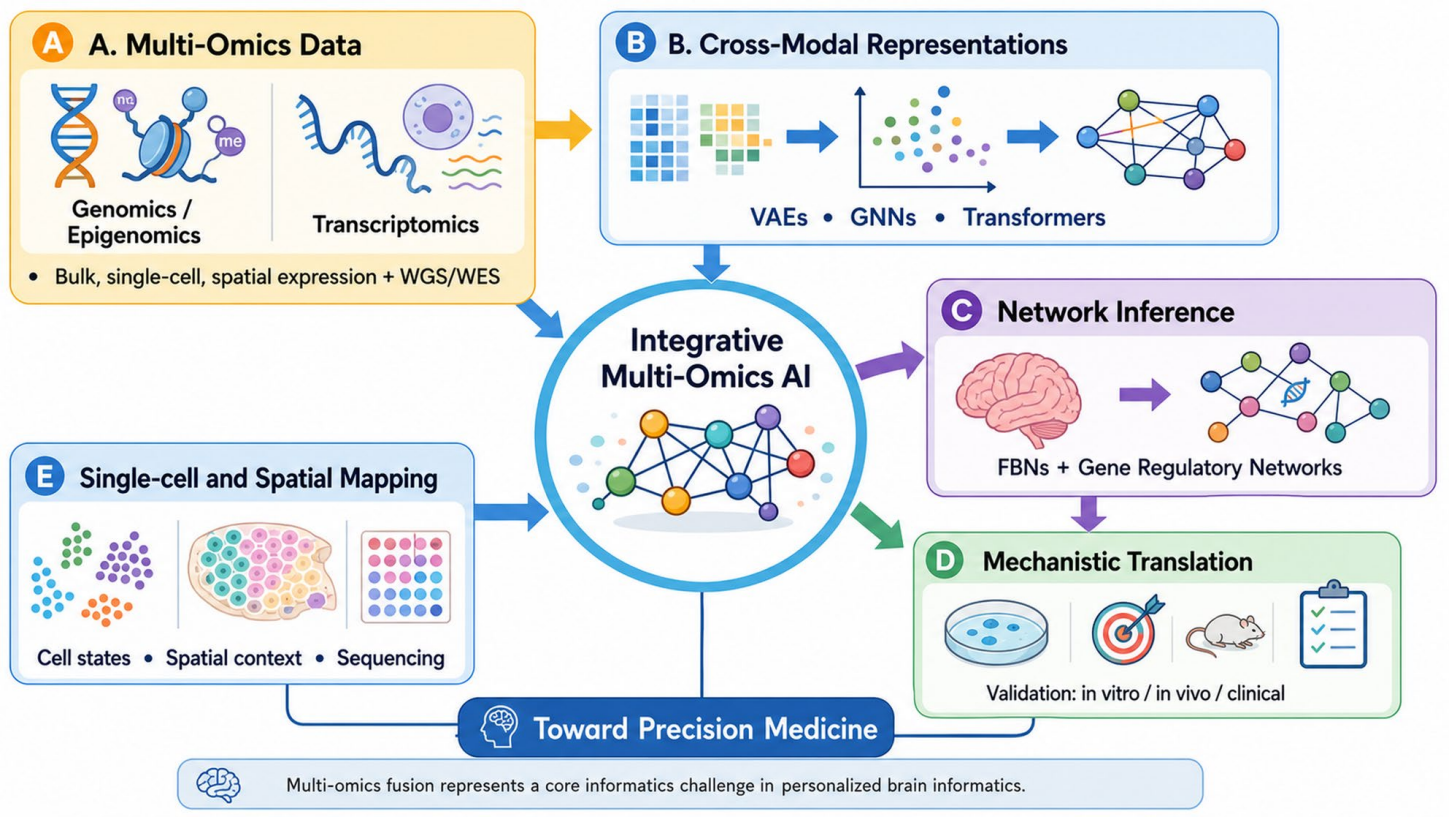
Multi-scale data fusion pipeline for brain network discovery. A brain informatics workflow integrating genomics/epigenomics and transcriptomics (bulk, single-cell, and spatial), cross-modal representation learning, and network inference to connect molecular regulation with functional brain networks. The pipeline highlights translation from integrative multi-omics AI to validated, precision-medicine hypotheses

**Fig. 2:**
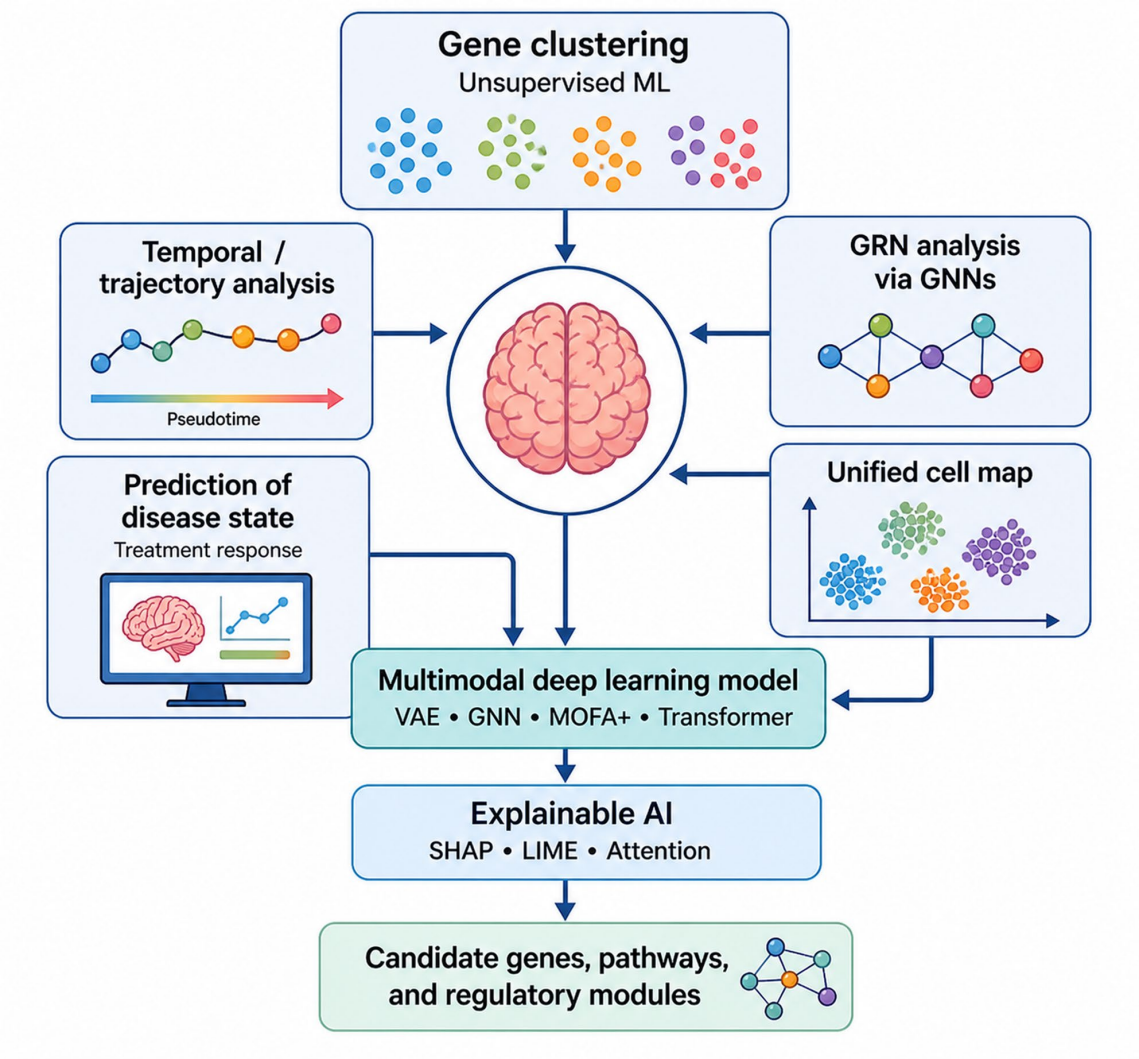
AI and machine learning modules in integrative neuro-omics. The workflow includes gene clustering, temporal/trajectory analysis, graph-based GRN modeling, multimodal deep learning, and explainable AI workflows that support mechanistic interpretation and hypothesis prioritization

We categorize current methodologies into four hierarchical layers that form a coherent discovery pipeline (Fig. 1), and these modules are further organized into the multimodal deep learning workflow summarized in Fig. 2:

1. *Representation learning*: Utilizing VAEs and manifold learning (UMAP/t-SNE) to transform high-dimensional, noisy data into compact latent embeddings while preserving biological structure.
2. *Cross-modal fusion and alignment*: Integrating heterogeneous modalities—such as genomics, epigenomics, and imaging—into shared latent spaces using multimodal transformers or Canonical Correlation Analysis (CCA).
3. *Network and causal inference*: Employing Graph Neural Networks (GNNs) and Bayesian frameworks to infer the regulatory edges between genes, cell types, and macroscopic brain regions.
4. *Dynamics and interpretability*: Utilizing temporal models (e.g., Hidden Markov Models or trajectory inference) to capture system evolution, followed by Explainable AI (XAI) to translate these predictions into mechanistic hypotheses.

### Comparative selection across omics modalities

Methodological choice is fundamentally driven by the constraints of the data modality, particularly the distinction between bulk-level signal, single-cell heterogeneity, and multimodal graph-based patient classification [15, 16]:

- *Bulk omics*: Prioritizes regularized statistical models and classical machine learning to maximize insights from larger sample sizes with lower resolution.
- *Single-cell omics*: Necessitates non-linear embedding techniques (VAEs/scVI) to address cellular heterogeneity and technical “dropouts.”
- *Spatial omics*: Demands “spatially aware” models, such as Graph Convolutional Networks, that treat physical tissue coordinates as an essential feature of the regulatory context.

**Table 2:** Comparative assessment of GRN inference routes

Technical route	Core principles	Primary application	Major bottleneck	Consensus
Statistical (WGCNA)	Linear correlation	Bulk screening	No directionality	Baseline standard
Bayesian inference	Probabilistic graphs	Targeted gene sets	Poor scalability	High interpretability but limited scalability
Graph AI (GNNs)	Message passing	Multi-modal fusion	Prior dependency	Emerging standard for graph-based integration
Regularized ML (LASSO / ElasticNet)	L1/L2 penalization	Biomarker selection from high-dimensional bulk omics	Class imbalance; feature redundancy	High-throughput screening baseline
SCENIC / GENIE3 / GRNBoost2	Regression + boosted trees on co-expression	Cell-type-specific GRN inference from scRNA-seq	Scalability; requires dense single-cell data	Community-standard GRN tools
Variational models (VAEs / scVI)	Latent variable generative models	Single-cell denoising and multi-batch integration	Interpretability of latent dimensions	De facto standard for scRNA-seq normalization
Foundation models (scGPT / Geneformer)	Pre-trained transformers on gene tokens	Cross-dataset zero-shot transfer; perturbation prediction	Compute cost; training data bias	Emerging frontier for pan-omics integration

### Hybrid modeling and practical constraints

**Fig. 3:**
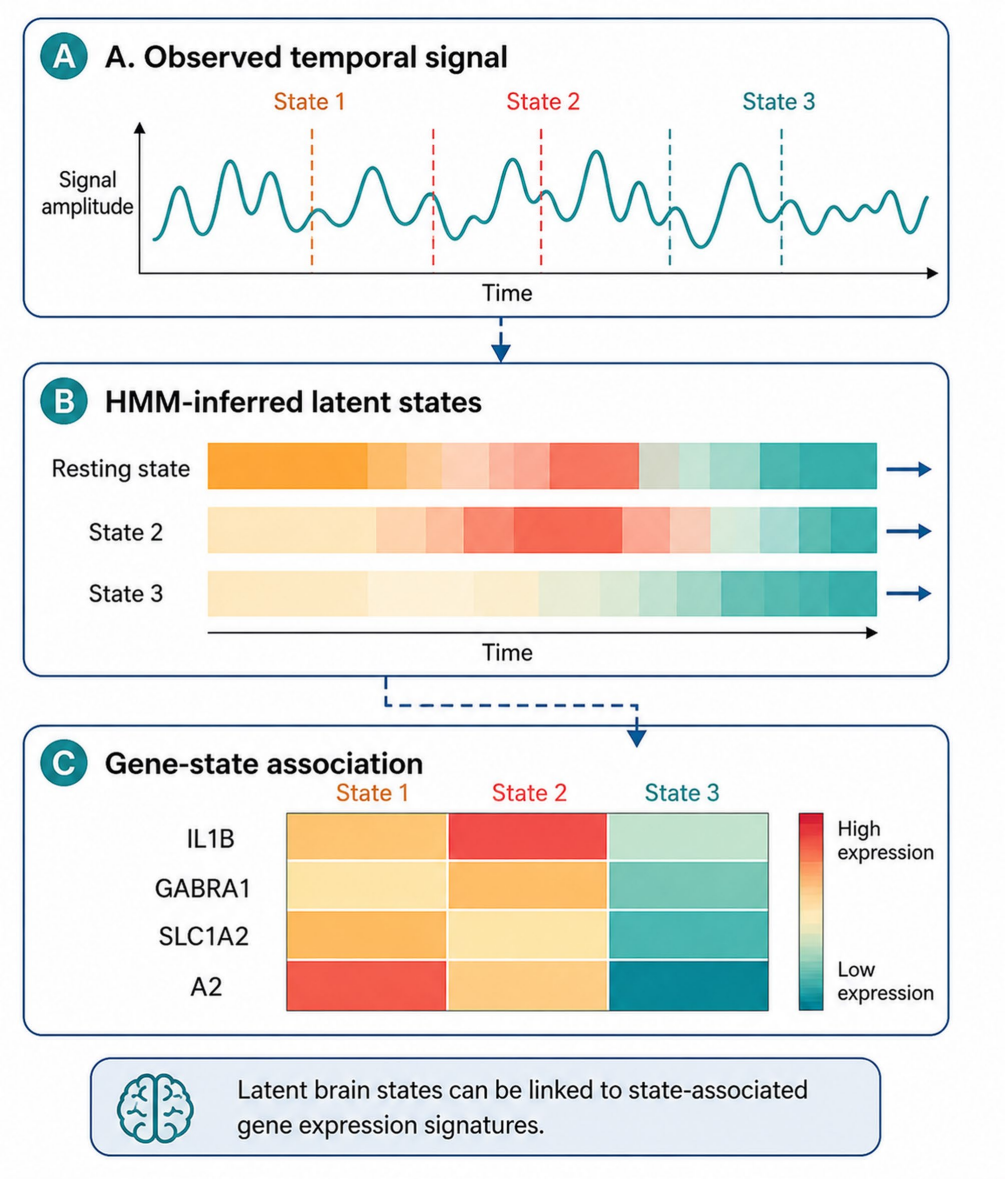
Temporal modeling of brain network dynamics with hidden Markov models. Time-resolved signals are segmented into discrete latent states, which can be linked to state-associated gene expression signatures (illustrative genes shown) to interpret molecular drivers of network reconfiguration

Superior biological insight is frequently achieved through hybrid strategies. For instance, pairing a VAE with a GNN allows for latent-space regulatory inference, while combining multimodal fusion with XAI facilitates the discovery of complex biomarkers in clinical cohorts. The selection of these hybrids must account for practical constraints: small sample sizes favor prior-driven Bayesian models, while longitudinal datasets require the integration of temporal modeling (HMMs/RNNs), as illustrated by latent-state and trajectory workflows in Fig. 3 [17–22].

## Cross-scale integration: linking molecules to brain networks

### Cross-scale modeling approaches

Beyond basic spatial alignment to anatomical atlases, three sophisticated informatics routes are emerging:

1. *Hierarchical modeling*: Integrating data across scales from the gene level to cell-type specific circuits, and finally to global functional hubs.
2. *Multi-resolution GNNs*: Jointly modeling molecular regulatory graphs and brain connectivity graphs to identify conserved motifs across scales.
3. *Cross-modal attention*: Utilizing Transformer-based architectures to weigh the influence of specific molecular markers on macroscopic imaging phenotypes.

### Technical bottlenecks and controversies

**Fig. 4:**
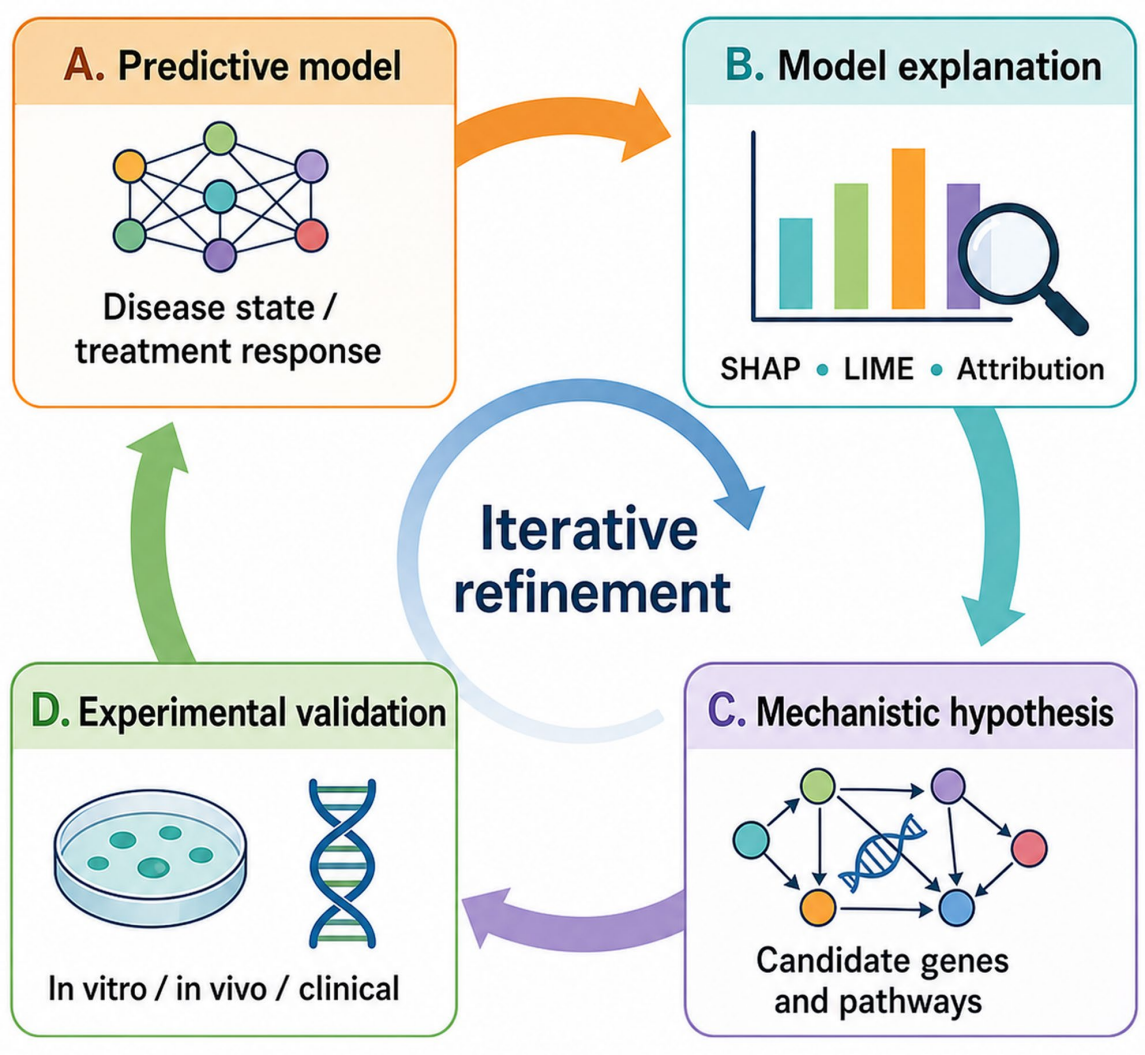
Explainable AI loop for mechanistic discovery in brain informatics. Model predictions are interpreted (e.g., SHAP/attribution), converted into mechanistic hypotheses, and iteratively refined through experimental and/or clinical validation

The primary challenge in cross-scale modeling is the resolution mismatch. A voxel in an fMRI scan typically aggregates thousands of cells, masking cell-type specific contributions to neural circuit stability. Furthermore, temporal discordance across modalities—where gene expression dynamics occur on a different time scale than functional blood-oxygen-level-dependent (BOLD) signals—complicates causal modeling. Bridging this gap may benefit from Neural Ordinary Differential Equations (NODEs), which can model the continuous temporal evolution of these biological layers. Interpretable biomarker selection and model-attribution strategies provide a practical route for converting such predictions into testable hypotheses (Fig. 4) [23–35].

## Application to neurological disorders

### Alzheimer’s disease (AD) and neurodegeneration

In AD, AI models have transitioned from descriptive pathology to identifying the regulatory drivers of neuro-inflammation. Deep learning applied to single-nucleus RNA-seq (snRNA-seq) has successfully characterized the transition of homeostatic microglia into *Disease-Associated Microglia (DAM)* [36]. By employing causal GNNs on these datasets, researchers have identified master transcription factors such as *SPI1* (PU.1)—that coordinate the switch toward pro-inflammatory states [36, 37]. Furthermore, AI-driven integration of PET imaging and transcriptomics has revealed that specific regional “vulnerability signatures” are dictated by the expression of synaptic maintenance genes, providing a molecular basis for the regional selectivity of tau pathology [37, 38].

### Schizophrenia and neurodevelopmental logic

Schizophrenia manifests as a disruption in the hierarchical organization of cortical circuits. AI-integrated multi-omics has revealed that risk variants identified in Genome-Wide Association Studies (GWAS) are significantly enriched in excitatory neuronal lineages during early mid-gestation. By utilizing *Representation Learning* to integrate Polygenic Risk Scores (PRS) with longitudinal fetal brain atlases, researchers have mapped how these variants disrupt synaptic pruning [39, 40]. Advanced trajectory modeling now predicts the deviation of neurodevelopmental paths years before clinical onset, highlighting the role of the MHC region and complement system in synaptic elimination [41, 42].

### Epilepsy and network hyperexcitability

The application of AI in epilepsy focuses on the interplay between transcriptional state and electrophysiological phenotype. Multi-view learning architectures have been used to link EEG-derived connectivity features with spatial transcriptomics in resected focal tissues. These models have highlighted the role of non-coding RNAs (ncRNAs) in the fine-tuned regulation of ion channel density. Specifically, HMM- and GNN-based approaches have identified localized “seizure hubs” where the disruption of GABAergic interneuron gene expression leads to runaway excitation, providing a roadmap for cell-type specific gene therapies [21].

## Field-wide challenges and controversies

### Interpretability vs. predictive power

The “Black Box” nature of deep learning remains a contentious issue in clinical brain informatics. While models may achieve high accuracy in diagnosing disease states, the lack of *mechanistic transparency* hinders the translation of AI findings into actionable drug targets. Emerging frameworks in *Mechanistic XAI* attempt to bridge this by enforcing biological constraints on the neural network’s hidden layers.

### The pseudotime validation gap

Reconstructing temporal trajectories (pseudotime) from static, cross-sectional snapshots of diverse donors is a standard practice in the field. However, critics argue this may fail to capture the idiosyncratic variability of individual disease progression. There is an urgent need for longitudinal, multi-omic datasets from a single cohort to validate these AI-generated “clock” models of brain aging.

### Over-harmonization and signal loss

Aggressive computational batch-correction (e.g., Harmony, LIGER) is necessary for large-scale integration but risks “over-smoothing” rare biological signals. This is particularly relevant in neuro-psychiatry, where subtle cell-state transitions may be dismissed as technical noise, a debate that remains central to the construction of unified brain cell atlases.

## The gut–brain axis: computational perspectives

The traditionally descriptive view of the gut–brain axis is evolving through quantitative systems biology. Current efforts focus on microbiome–metabolome–transcriptome fusion using physics-informed neural networks (PINNs). These models simulate the transport kinetics of microbial metabolites (e.g., short-chain fatty acids) across the blood–brain barrier, quantifying their role as epigenetic modifiers—specifically through HDAC inhibition and histone acetylation—in the prefrontal cortex and hippocampus [43–47]. Complementary approaches include graph-based host–microbe regulatory network inference using GNNs applied to paired metagenomics and host transcriptomics, dynamic Bayesian models for longitudinal microbiome–brain state coupling, and variational autoencoders for latent representation of multi-kingdom regulatory modules. Together, these mechanistic machine learning frameworks move the gut–brain axis from descriptive biology toward testable, systems-level computational models.

## Targeted future directions

To resolve current bottlenecks, the field must prioritize biologically grounded computational innovation. Future research should move beyond generic AI adoption toward targeted trajectories that reconcile molecular dynamics with systems-level neuroscience.

### Causal AI and unsupervised regulatory discovery

Current regulatory network inference relies heavily on human-curated databases as “ground truth” priors. However, these databases are often biased toward well-studied pathways, potentially masking novel regulatory hubs. Future research must prioritize the development of *Unsupervised Causal Discovery* algorithms that can identify de novo regulatory motifs directly from single-cell multiome data. By integrating structural equation modeling (SEM) with GNNs, these models could differentiate between “passenger” transcriptional changes and “driver” master regulators, facilitating the identification of therapeutic targets that are robust to cellular context.

### Cross-scale digital twins for precision neurology

The integration of longitudinal omics and macro-scale neuroimaging presents an opportunity to construct person-specific *Digital Twins*. These in silico models would simulate the progression of neurodegenerative or neurodevelopmental disorders based on an individual’s unique genetic and connectivity profile. By utilizing *Physics-Informed Neural Networks (PINNs)*, these twins could predict how specific pharmacological interventions alter the transcriptional landscape of a cortical circuit and, subsequently, its functional output. Such models could support in silico treatment prioritization and dosage optimization before patient administration.

### Federated learning and data sovereignty in rare diseases

Large-scale AI models require massive datasets, which are often unavailable for rare neurological conditions or specific neuro-psychiatric subtypes. *Federated Learning* offers a feasible path forward by allowing models to be trained across multiple institutions without moving sensitive patient data. This decentralized approach maintains data sovereignty and privacy while aggregating the statistical power needed to identify rare variants. Future frameworks should focus on “Heterogeneous Federated Learning,” capable of harmonizing models across sites with varying imaging protocols and omics platforms.

### Foundation models for brain resilience and plasticity

While most neuro-informatics research focuses on disease states, there is a significant need for models that decode the molecular grammar of *Resilience*. Scaling genomic foundation models (e.g., scGPT) to include individuals who remain phenotypically healthy despite high polygenic risk could reveal novel “protective” gene expression signatures. Integrating these models with *Spatial–Temporal Multimodal Modeling* will allow us to understand how the brain’s compensatory plasticity is maintained at the molecular level, offering a blueprint for therapies aimed at enhancing cognitive reserve rather than merely reducing pathology.

### Clinically interpretable decision-support systems

Finally, the transition from research to clinic requires AI systems that offer actionable interpretability. Future directions should emphasize the development of “human-in-the-loop” informatics tools, where XAI provides clinicians with not just a prediction (e.g., “High risk of AD”) but a quantifiable mechanistic path (e.g., “Predicted breakdown of microglia-neuron crosstalk via SPI1 hyper-regulation”). This transition will require standardized coordinate systems, spatial transcriptomic benchmarks, robust batch-correction strategies, and cross-scale graph models that serve as a common language for biologists, data scientists, and clinicians alike [48–55].

## Supplementary Information

Below is the link to the electronic supplementary material.

Supplementary Material 1

## Data Availability

No datasets were generated or analysed during the current study.

## Declarations

Ethics declaration: Not applicable.

## Abbreviations

MEG: Magnetoencephalography
EEG: Electroencephalography
fMRI: Functional magnetic resonance imaging
ST: Spatial transcriptomics
ChIP-seq: Chromatin immunoprecipitation sequencing
ATAC-seq: Assay for transposase-accessible chromatin with sequencing
scRNA-seq: Single-cell RNA sequencing
GRN: Gene regulatory network
FBN: Functional brain network
HMM: Hidden Markov model
DTW: Dynamic time warping
UMAP: Uniform manifold approximation and projection
ARIMA: Autoregressive integrated moving average
MRI: Magnetic resonance imaging
PCA: Principal component analysis
VAE: Variational autoencoder
GNN: Graph neural network
XAI: Explainable artificial intelligence
ML: Machine learning
AI: Artificial intelligence
