# Supplementary material for: Decoding neuronal gene expression: integrative insights from omics and AI
Source: Brain Inform. 2026 Jul 21;13(1):36. doi: 10.1186/s40708-026-00323-z (PMC13481912; doi:10.1186/s40708-026-00323-z)
Supplement: Supplementary file 1 [file 40708_2026_323_MOESM1_ESM.docx]

# Additional file 1: Supplementary Table S1. Key datasets, computational techniques, and integration challenges in AI-driven brain informatics.

| **SECTION A: Key Datasets** | | | | |
| --- | --- | --- | --- | --- |
| **Dataset Name** | **Data Type** | **Purpose** | **Scale & Resolution** | **Key Application in Brain Informatics** |
| **Allen Brain Observatory / Atlas (de Vries et al., 2023)** | Imaging, transcriptomics | Map neuronal activity and gene expression by region | 100,000+ neurons, cellular resolution | Linking gene activity to neural firing; cortical structure–function relationships |
| **Tabula Muris (Tabula Muris Consortium, 2020)** | Single-cell RNA-seq | Cluster neuronal and immune subtypes | ~100,000 cells, single-cell resolution | Exploring immune–neuronal interactions; cross-species comparisons |
| **Human Cell Atlas (Rood et al., 2025; Rozenblatt-Rosen et al., 2017)** | Multi-omics | Integrate transcriptomics and epigenomics across tissues | 10M+ cells, single-cell & spatial resolution | Tracking neurodevelopmental trajectories; cell-type atlas construction |
| **Roadmap Epigenomics (Satterlee et al., 2019)** | Epigenomics (histone ChIP-seq, ATAC-seq) | Map histone modifications across tissues and cell types | Genome-wide, high-resolution | Identifying histone plasticity markers; regulatory element annotation |
| **PsychENCODE** | Bulk and single-cell RNA-seq, ATAC-seq, Hi-C | Decode gene regulation in neuropsychiatric disorders | Multiple brain regions, multi-cohort | Linking GWAS variants to gene expression in schizophrenia and ASD |
| **ADNI (Alzheimer's Disease Neuroimaging Initiative)** | MRI, PET, CSF biomarkers, genomics | Stage AD progression and identify biomarkers | Longitudinal, 1,500+ participants | Multimodal AI for early AD diagnosis and progression prediction |
| **AMP-AD / ROSMAP** | RNA-seq, proteomics, methylation, clinical | Characterize molecular drivers of AD | Post-mortem multi-region brain tissue | Identifying transcription factors and GRN hubs in neurodegeneration |
| **BICCN / BICAN** | scRNA-seq, ATAC-seq, MERFISH spatial | Comprehensive mammalian brain cell atlas | Whole brain, single-cell & spatial resolution | Defining cell types and regulatory programs across cortical layers |
| **GTEx Brain Tissues** | Bulk RNA-seq across brain regions | Baseline tissue-level gene expression | Genome-wide, ~50 brain regions | eQTL mapping; tissue-specific expression baselines for disease studies |
| **ENCODE / EpiMap** | ChIP-seq, ATAC-seq, RNA-seq, Hi-C | Annotate regulatory elements genome-wide | Genome-wide, multi-tissue | Prioritizing non-coding variants; enhancer-promoter interaction maps |
| **Allen Human Brain Atlas** | ISH, microarray, RNA-seq | Spatial gene expression across the entire human brain | Anatomically registered, ~1,000 brain structures | Brain-region-specific expression profiles; neuroanatomical gene mapping |

| **SECTION B: Computational Techniques** | | | | |
| --- | --- | --- | --- | --- |
| **Methodology** | **Function** | **Example Use Case** | **Dataset(s) Used** | **Strength** |
| **LASSO / ElasticNet Regression** | Feature selection via L1/L2 penalization | Identifying early Alzheimer's biomarkers from multi-omics panels | ADNI, NACC, AMP-AD cohorts | Selects sparse, interpretable gene markers; computationally efficient |
| **WGCNA** | Weighted gene co-expression network construction | Identifying disease-associated co-expression modules in bulk RNA-seq | GTEx, ROSMAP, PsychENCODE | Unsupervised; identifies hub genes; well-validated community standard |
| **SCENIC / GENIE3 / GRNBoost2** | GRN inference via regression and boosted trees | Cell-type-specific transcription factor regulon discovery from scRNA-seq | Human Cell Atlas, BICCN brain atlases | Community-standard single-cell GRN tools; supports regulon scoring |
| **MOFA+ (Multi-Omics Factor Analysis)** | Latent factor decomposition across omics layers | Decomposing shared and modality-specific variation in multi-omics data | AMP-AD, PsychENCODE, custom cohorts | Interpretable latent factors; handles missing modalities |
| **scVI / totalVI / MultiVI** | Variational autoencoders for single-cell integration | Batch correction and normalization across single-cell datasets | Human Cell Atlas, BICCN, 10x Genomics datasets | De facto standard for scRNA-seq normalization and integration |
| **Seurat / WNN (Weighted Nearest Neighbor)** | Multimodal single-cell data integration | Combining RNA-seq and ATAC-seq for joint cell clustering | 10x Multiome datasets, BICCN | Widely adopted; robust for paired multiome integration |
| **Harmony / LIGER** | Batch correction and cross-dataset integration | Harmonizing brain atlas data across institutions and platforms | GTEx, ROSMAP, Allen Brain Atlas | Fast; preserves biological variation while removing technical noise |
| **Graph Neural Networks (GNNs)** | Message-passing on biological graphs for network modeling | Inferring causal regulatory pathways; brain connectivity modeling | Benchmark GRN datasets, fMRI connectivity graphs | Captures complex multi-gene interactions; scalable to multimodal fusion |
| **SHAP (SHapley Additive exPlanations)** | Feature attribution via game-theoretic decomposition | Identifying key transcription factors driving neurological disease predictions | AD cohorts, transcriptomic classifiers | Model-agnostic interpretability; supports biological hypothesis generation |
| **Hidden Markov Models (HMMs)** | Latent state inference from time-series data | Decoding dynamic brain states from EEG/fMRI and linking to gene expression | Epilepsy HMM studies, bipolar disorder datasets | Recovers discrete computational brain states; principled temporal modeling |
| **Trajectory Inference (Monocle, Palantir, scVelo)** | Pseudotime and RNA velocity for developmental ordering | Reconstructing neurodevelopmental trajectories from scRNA-seq | Human Cell Atlas, BICCN developmental datasets | Reveals differentiation paths; identifies key regulatory transitions |
| **Federated Learning** | Distributed model training without centralizing patient data | Training AD or rare-disease classifiers across multiple hospital sites | Multi-site clinical cohorts (ADNI, ROSMAP, local EHR) | Preserves data privacy; enables rare-disease power aggregation |
| **scGPT / Geneformer (Foundation Models)** | Large-scale pre-trained Transformers on gene expression tokens | Zero-shot cell annotation; perturbation response prediction | Large-scale single-cell atlases (CELLxGENE, HCA) | Generalizable representations; minimal fine-tuning for new tasks |

| **SECTION C: Integration Challenges in Multi-Omics Brain Informatics** | | | |
| --- | --- | --- | --- |
| **Challenge** | **Issue Description** | **Suggested Approach** | **Impact on Research** |
| **Batch Effects** | Systematic technical variation across labs, platforms, or sequencing runs confounds biological signals in multi-cohort integration | Standardized data pipelines; Harmony, LIGER, or scVI-based batch correction; reference material ratio normalization (Yu et al., 2023) | Improves cross-study reproducibility and validity of downstream analysis |
| **Data Sparsity** | Incomplete or zero-inflated feature representation in single-cell data (dropout) leads to biased imputation and inflated false-discovery rates | Zero-inflated negative binomial models; robust imputation methods (MAGIC, DeepImpute); variational autoencoders | Enables detection of rare neuronal subtypes and low-abundance regulatory states |
| **Limited Interpretability of Deep Learning Models** | Black-box neural networks produce accurate predictions but lack mechanistic transparency essential for clinical adoption and biological insight | Implementation of explainable AI frameworks (SHAP, LIME, integrated gradients); mechanistic XAI with biological constraints on hidden layers | Enhances AI-driven biomarker validation and clinical trust in model outputs |
| **Resolution Mismatch Across Scales** | Molecular data (single-cell resolution) and neuroimaging data (voxel-level) operate at incompatible spatiotemporal scales, complicating causal cross-scale inference | Hierarchical modeling; multi-resolution GNNs; cross-modal attention transformers; pseudo-bulk aggregation strategies | Critical bottleneck for translating molecular findings to brain network phenotypes |
| **Over-Harmonization / Signal Loss** | Aggressive batch correction may erase genuine rare biological variation, particularly subtle cell-state transitions in neuropsychiatric conditions | Benchmarking integration methods against ground-truth cell-state markers; controlled normalization with preserved rare cluster detection | Risks erasing disease-relevant subtypes from unified brain cell atlases |
| **Validation Gap in Pseudotime / Trajectory Models** | Temporal trajectories reconstructed from cross-sectional static snapshots may not reflect true longitudinal disease progression in individual patients | Longitudinal multi-omic cohorts; orthogonal validation with CRISPR perturbation data; comparison against RNA velocity ground-truth | Limits confidence in AI-generated aging clocks and neurodevelopmental trajectory models |
| **Computational Scalability** | Large-scale foundation models and multi-omics pipelines demand extensive GPU compute and memory, limiting accessibility for many research groups | Efficient approximations (flash attention, sparse transformers); federated learning; cloud-based distributed training frameworks | Access barriers slow adoption in resource-limited settings and reduce dataset diversity |
| **Population Bias and Generalizability** | Genomic and transcriptomic datasets are disproportionately derived from European-ancestry individuals, reducing AI model generalizability across diverse populations | Actively diversifying data collection (NIAGADS, ADSP); transfer learning and domain adaptation; federated learning across diverse sites | Necessary for equitable precision medicine and globally applicable clinical tools |

*Abbreviations: AD, Alzheimer's disease; ATAC-seq, assay for transposase-accessible chromatin with sequencing; BICCN, Brain Initiative Cell Census Network; BICAN, Brain Initiative Cell Atlas Network; EHR, electronic health records; eQTL, expression quantitative trait locus; GRN, gene regulatory network; GWAS, genome-wide association study; HCA, Human Cell Atlas; HMM, hidden Markov model; ISH, in situ hybridization; LASSO, least absolute shrinkage and selection operator; MERFISH, multiplexed error-robust fluorescence in situ hybridization; PET, positron emission tomography; scRNA-seq, single-cell RNA sequencing; SHAP, SHapley Additive exPlanations; WGCNA, weighted gene co-expression network analysis; WNN, weighted nearest neighbor.*
